# mSphere of Influence: Hooray for HaloTag! A novel tool to study autophagy in mammalian cells

**DOI:** 10.1128/msphere.00482-23

**Published:** 2023-10-31

**Authors:** Laura Cheney

**Affiliations:** 1 Department of Medicine, Albert Einstein College of Medicine, New York, New York, USA; 2 Department of Pathology, Albert Einstein College of Medicine, New York, New York, USA; University of Michigan, Ann Arbor, Michigan, USA

**Keywords:** autophagy, Atg8/LC3 processing assay, mitophagy, HaloTag, HIV associated neurocognitive impairment, antiretroviral therapy

## Abstract

Laura Cheney works at the crossroads of HIV and autophagy, a critical biological process for cellular homeostasis, to understand more completely the pathogenesis of HIV-associated neurocognitive impairment. In this mSphere of Influence article, she reflects on how “A pulse-chasable reporter processing assay for mammalian autophagic flux with HaloTag” by Willa Wen-You Yim, Hayashi Yamamoto, and Noboru Mizushima (eLife 11:e78923, 2022, https://doi.org/10.7554/eLife.78923) expands the tools for studying autophagy and inspired her to use this technology to develop a reporter to study autophagy of mitochondria, termed mitophagy, to further her own research goals.

## COMMENTARY

Autophagy, Greek for “self-eating,” is an evolutionarily conserved, highly regulated, and complex intracellular process for degrading subcellular components in a lysosome-dependent manner. Autophagy maintains cellular homeostasis by providing building blocks for energy and eliminating damaged/dysfunctional organelles and toxic protein aggregates. Think of it as a garbage disposal and recycling program that is essential for cell and, by extension, organism survival. Autophagy occurs constitutively and is upregulated by a variety of stimuli that perturb homeostasis. When dysregulated, it contributes to many diseases including cancer and neurodegeneration (1). The ability to measure autophagy sensitively and accurately is fundamental to understanding how changes in autophagy relate to human health and disease. This commentary focuses on an exciting and innovative new tool to measure autophagy in mammalian cells, developed by Yim et al. as reported in “A pulse-chasable reporter processing assay for mammalian autophagic flux with HaloTag” (2). Their tool inspired me to create a custom protein tag to further my studies on the contribution of autophagic processes in astrocytes, central nervous system (CNS) cells vital for neuronal health, to HIV-associated neurocognitive impairment (HIV-NCI). HIV-NCI is a waxing/waning cognitive dysfunction affecting many people with HIV despite viral suppression with antiretroviral therapy (ART) (3). It decreases the quality of life and is a risk factor for mortality (4, 5). The pathogenesis is incompletely characterized, and autophagy dysregulation may contribute.

Autophagy is a multi-step process involving a specialized double-membrane vesicle termed autophagosome (APG). Intracellular cargo destined for degradation is sequestered inside APG. Cargo-loaded APG fuse with lysosomes after which the luminal contents of APG are degraded. From start to finish, this is called autophagic flux. Flux is measured most commonly with assays that detect autophagy-related protein 8 (Atg8; yeast) or microtubule-associated protein light chain 3 (LC3; mammals) (6). ATG8/LC3 studs the outer and inner APG membranes, remaining associated with APG throughout flux. The amount of Atg8/LC3 correlates with the number of APG present at any given time, thus indicating autophagic activity. Yeast autophagy is routinely monitored with the green fluorescent protein (GFP)-Atg8 processing assay. The keys to this assay’s ability to measure autophagy sensitively and accurately are as follows. (i) GFP is resistant to yeast vacuole proteases, accumulating in the vacuole as autophagy progresses. Flux is determined by detection of free GFP signal rather than by signal loss. (ii) Results are dependent on the delivery of GFP-Atg8 to the vacuole to generate free GFP, thus accurately reflecting autophagic activity. (iii) Lysosome inhibitors are not required to interpret results. Inhibitors can affect LC3 processing, are toxic, and can have off-target effects, thus confounding results. Rarely has an equivalent assay been successfully performed in mammalian cells (7, 8). This is because GFP is degraded by proteases in mammalian lysosomes, leaving little free GFP left to measure once APG fuse with lysosomes. The solution isn’t as simple as replacing GFP with red fluorescent protein (RFP), which is resistant to proteases. RFP accumulates in lysosomes due to basal autophagy, thus obscuring changes in RFP that would result from changes in flux. Other flux assays for mammalian cells have been developed, but these require lysosome inhibitors, are of low sensitivity, or are too complex to perform or interpret accurately (6). Yim et al. took advantage of the HaloTag protein tagging system to develop a processing assay for mammalian cells using a novel tool: Halo-LC3.

HaloTag is a modified bacterial haloalkane dehalogenase that forms covalent bonds with synthetic ligands that contain chloroalkane linkers. The covalent bond formed between HaloTag and linker occurs naturally and rapidly and is highly specific and protease resistant. The linker can be attached to various molecules that allow for detection by different modalities (9). Yim et al. saw great potential for HaloTag to fill the need for a mammalian LC3 processing assay. They developed a plasmid that genetically fused HaloTag to LC3 (Halo-LC3), which was stably expressed in HeLa cells (2). These cells were incubated with a red fluorescent ligand, tetramethylrhodamine (TMR), containing the chloroalkane linker, and then serum starved to induce flux. By immunoblotting for Halo, they show an increased amount of Halo with autophagy induction, similarly to that for GFP in the yeast GFP-Atg8 processing assay. In-gel fluorescence imaging of TMR corroborated these results. Their rigorous experiments confirmed that (i) Halo-LC3 localized to the appropriate autophagic structures, including lysosomes, (ii) HaloTag was resistant to lysosomal proteases when in the presence of ligand, (iii) they could sensitively measure autophagic flux with a wide linear range without needing lysosome inhibitors, and (iv) results were fully dependent on autophagy (2). Yim et al. succeeded in developing a processing assay for mammalian cells that had all of the benefits of the yeast GFP-Atg8 assay. Moreover, they show their tool is easily adapted for fluorescent microscopy or flow cytometry, as well as for studying other types of autophagy by simply exchanging LC3 for a different protein of interest involved in autophagy (2).

While many papers have influenced how I perform autophagy studies (6), this important paper inspired me to develop my own tool to study mitophagy, selective autophagic degradation of mitochondria. Mitophagy clears damaged and dysfunctional mitochondria from the cell and is vital for cell homeostasis (10). My current studies are focused on how antiretroviral drugs impact mitophagy in human astrocytes as a possible contribution to HIV-NCI pathogenesis. Antiretroviral drugs impact autophagy (11), but little is known on how ART affects mitophagy in astrocytes, cells critical for maintaining CNS homeostasis. I was attempting to measure mitophagy in ART-treated astrocytes using a dual-fluorescent mitophagy reporter. However, I had difficulty detecting fluorescence changes, and the reporter was toxic to cells over the course of longer experiments. After reading the paper by Yim et al., I was inspired to create a custom HaloTag that would label mitochondria and is adaptable for measuring mitophagy by multiple modalities. I feel confident my new mitophagy tool, based on the innovative Halo-LC3 developed by Yim et al., will help me accomplish my research goals. Understanding the impacts of ART on astrocyte mitophagy will further our understanding of HIV-NCI pathogenesis and could ultimately support development of therapies to mitigate or prevent HIV-NCI.

## References

[1] Mizushima N, Levine B, Cuervo AM, Klionsky DJ. 2008. Autophagy fights disease through cellular self-digestion. Nature 451:1069–1075. doi:10.1038/nature06639 18305538 PMC2670399

[2] Yim WW-Y, Yamamoto H, Mizushima N. 2022. A pulse-chasable reporter processing assay for mammalian autophagic flux with HaloTag. Elife 11:e78923. doi:10.7554/eLife.78923 35938926 PMC9385206

[3] Gott C, Gates T, Dermody N, Brew BJ, Cysique LA. 2017. Cognitive change trajectories in virally suppressed HIV-infected individuals indicate high prevalence of disease activity. PLoS One 12:e0171887. doi:10.1371/journal.pone.0171887 28264037 PMC5338778

[4] Jones JD, Kuhn T, Levine A, Sacktor N, Munro CA, Teplin LA, D’Souza G, Martin EM, Becker JT, Miller EN, Hinkin CH. 2019. Changes in cognition precede changes in HRQoL among HIV+ males: longitudinal analysis of the multicenter AIDS cohort study. Neuropsychology 33:370–378. doi:10.1037/neu0000530 30816783 PMC6666308

[5] Patel S, Parikh NU, Aalinkeel R, Reynolds JL, Dmello R, Schwartz SA, Mahajan SD. 2018. United States national trends in mortality length of stay (LOS) and associated costs of cognitive impairment in HIV population from 2005 to 2014. AIDS Behav 22:3198–3208. doi:10.1007/s10461-018-2128-z 29705930

[6] Klionsky DJ, Abdel-Aziz AK, Abdelfatah S, Abdellatif M, Abdoli A, Abel S, Abeliovich H, Abildgaard MH, Abudu YP, Acevedo-Arozena A, Adamopoulos IE, Adeli K, Adolph TE, Adornetto A, Aflaki E, Agam G, Agarwal A, Aggarwal BB, Agnello M, Agostinis P, Agrewala JN, Agrotis A, Aguilar PV, Ahmad ST, Ahmed ZM, Ahumada-Castro U, Aits S, Aizawa S, Akkoc Y, Akoumianaki T, Akpinar HA, Al-Abd AM, Al-Akra L, Al-Gharaibeh A, Alaoui-Jamali MA, Alberti S, Alcocer-Gomez E, Alessandri C, Ali M, Alim Al-Bari MA, Aliwaini S, Alizadeh J, Almacellas E, Almasan A, Alonso A, Alonso GD, Altan-Bonnet N, Altieri DC, Alvarez EMC, Alves S. 2021. Guidelines for the use and interpretation of assays for monitoring autophagy. In Autophagy. doi:10.1080/15548627.2020.1797280:1382.

[7] Gao W, Ding WX, Stolz DB, Yin XM. 2008. Induction of macroautophagy by exogenously introduced calcium. Autophagy 4:754–761. doi:10.4161/auto.6360 18560273 PMC2696695

[8] Hosokawa N, Hara Y, Mizushima N. 2006. Generation of cell lines with tetracycline-regulated autophagy and a role for autophagy in controlling cell size. FEBS Lett 580:2623–2629. doi:10.1016/j.febslet.2006.04.008 16647067

[9] Los GV, Encell LP, McDougall MG, Hartzell DD, Karassina N, Zimprich C, Wood MG, Learish R, Ohana RF, Urh M, Simpson D, Mendez J, Zimmerman K, Otto P, Vidugiris G, Zhu J, Darzins A, Klaubert DH, Bulleit RF, Wood KV. 2008. HaloTag: a novel protein labeling technology for cell imaging and protein analysis. ACS Chem Biol 3:373–382. doi:10.1021/cb800025k 18533659

[10] Scrivo A, Bourdenx M, Pampliega O, Cuervo AM. 2018. Selective autophagy as a potential therapeutic target for neurodegenerative disorders. Lancet Neurol 17:802–815. doi:10.1016/S1474-4422(18)30238-2 30129476 PMC6359907

[11] Cheney L, Guzik H, Macaluso FP, Macian F, Cuervo AM, Berman JW. 2020. HIV Nef and antiretroviral therapy have an inhibitory effect on autophagy in human astrocytes that may contribute to HIV-associated neurocognitive disorders. Cells 9:1426. doi:10.3390/cells9061426 32526847 PMC7349791

